# One-by-One or All-at-Once? Self-Reporting Policies and Dishonesty

**DOI:** 10.3389/fpsyg.2016.00113

**Published:** 2016-02-17

**Authors:** Rainer M. Rilke, Amos Schurr, Rachel Barkan, Shaul Shalvi

**Affiliations:** ^1^Department of Corporate Development and Business Ethics, University of CologneCologne, Germany; ^2^Department of Business Administration, Ben Gurion University of the NegevBeer-Sheva, Israel; ^3^CREED, Faculty of Economics and Business, University of AmsterdamAmsterdam, Netherlands; ^4^Department of Psychology, Ben Gurion University of the NegevBeer-Sheva, Israel

**Keywords:** dishonesty, behavioral ethics, monitoring, trust, lie aversion, justifications, organizational policy

## Abstract

Organizational monitoring relies frequently on self-reports (e.g., work hours, progress reports, travel expenses). A “one-by-one” policy requires employees to submit a series of reports (e.g., daily or itemized reports). An “all-at-once” policy requires an overall report (e.g., an annual or an overview report). Both policies use people's self-reports to determine their pay, and both allow people to inflate their reports to get higher incentives, that is, to cheat. Objectively, people can cheat to the same extent under both reporting policies. However, the two policies differ in that the segmented one-by-one policy signals closer monitoring than the all-at-once policy. We suggest here that lie aversion may have a paradoxical effect on closer monitoring and lead people to cheat more. Specifically, reporting a series of segmented units of performance (allowing small lies) should lead to more cheating than a one-shot report of overall performance (that require one larger lie). Two surveys indicated that while people perceive the all-at-once policy as more trusting, they still expected people would be equally likely to cheat in both policies. An experiment tested the effects of the two reporting policies on cheating. The findings showed that contrary to the participants' intuition, but in line with research on lie aversion, the one-by-one policy resulted in more cheating than the all-at-once policy. Implications for future research and organization policy are discussed.

## Introduction

Honesty and trust are cornerstones of organizational success. For instance, Watson Wyatt's Work USA 2002 survey indicated the 3-year total return to shareholders was almost three times higher in companies characterized by high levels of honesty and trust than in companies characterized by low levels of honesty and trust. Decades of organizational research back up this example, teaching us that honesty and trust are important to both employers and employees (McGregor, 1960; Jones, 1991; Murphy, 1993; Moore and Gino, 2015). Honesty and trust are associated with higher levels of cooperation, better performance, proactive actions, effective management, and organizational growth (e.g., Jones and George, 1998; De Cremer et al., 2001; Dirks and Ferrin, 2001; Tyler, 2003; Cook et al., 2005). In addition, research suggests that ethical behavior elicits intrinsic incentives such as satisfaction and a sense of self-dignity (Peer et al., 2014; Moore and Gino, 2015).

Clearly, maintaining high ethical standards and fostering trust is advantageous for organizations. However, the combination of honesty and trust is easier preached than achieved. In fact, research suggests a counter-productive tradeoff exists between the two constructs. Specifically, close monitoring and increased enforcement are effective means to increase honesty, but are considered detrimental to trust (Kirchler, 2007; Kirchler et al., 2008). Lowering the levels of monitoring may boost trust, but—as rational economic analysis and behavioral research show—such leniency frequently leads to a gradual deterioration of ethical standards and an increase in dishonest behavior (Becker, 1974; Kirchler, 2007; Kirchler et al., 2008; Gino and Bazerman, 2009). Thus, designing an organizational environment that encourages both honest behavior and trust is a challenge, and choosing optimal policies is not straightforward.

A common organizational solution lets employees monitor and report their own performance. Taking self-reports at face value, organizations signal that they trust their employees and expect honest reports of true performance in return. An optimistic view suggests that trusting policies can foster loyalty, increase productivity, boost satisfaction, and reduce turnover (e.g., Shockley-Zalabak et al., 2000; Parker et al., 2006). A more pessimistic take is that self-report policies may tempt employees to inflate their performance levels and cheat at the expense of the organization.

Specific reporting procedures and especially the resolution level of self-reports, reflect these different takes on self-reported performance. Some organizations require regular segmented reports (e.g., hours worked, number of items produced, quality control measures; here dubbed the “one-by-one policy”). Other organizations require overall reports (e.g., summarizing a work project, expense reimbursements; here dubbed the “all-at-once” policy). By its nature, the micro-management approach of the one-by-one policy provides a higher level of monitoring compared to the relatively more macro-management approach of the all-at-once policy. In the current work, we compare the two reporting policies, and examine whether the difference between the policies is merely a semantic nuance, or whether it leads to different responses and affects the level of honesty.

Objectively, people can exploit both policies to the same extent. For example, consider a company representative reporting the number of customers that were interested in a new service he offered. Suppose that on 5 consecutive days, the numbers of interested customers were 1, 2, 3, 4, and 5. The representative can inflate daily reports of performance (e.g., reporting 2, 3, 4, 5, and 6) or inflate an overall report (e.g., report 20 instead of 15). Thus, from a rational economic analysis the two policies should lead to similar levels of cheating. However, Research on lie aversion shows that people justify small lies more easily than big lies. To avoid the adversity of being “real” liars, people tend to restrict their own dishonesty to a level they can justify (e.g., Ayal et al., 2015; Shalvi et al., 2015). Thus, rather than cheating to the maximal extent (for maximal profit), people tend to cheat “only by a little” to benefit from cheating but still maintain a sense of morality (Mazar et al., 2008; Shalvi et al., 2011a). Empirical evidence indicates that even when participants are guaranteed they will not be caught, punished, or even identified as cheaters, they still exhibit lie aversion (Gneezy, 2005; Lundquist et al., 2009; Shalvi et al., 2011b; Hilbig and Hessler, 2013; Weisel and Shalvi, 2015). Applying the reasoning of lie aversion to the abovementioned hypothetical example suggests the company's representative will feel more comfortable telling five small lies than one big lie. Accordingly, people will cheat more when they submit a series of small reports in the one-by-one policy, and will restrain themselves when they submit a single overall report in the all-at-once policy. This is the possibility we test here.

Interestingly, there are two ways in which the mean level of cheating can be higher in the one-by-one compared to the all-in-once setting. One option is that more people may be tempted to lie just a bit in the one-by-one setting compared to the all-at-once setting. If this is true, we should observe that the distribution of reported performance in the one-by-one distribution is shifted to the right indicating more people reported higher outcomes. A second option is that a similar proportion of people will lie in both settings, but lies will be larger in the one-by-one policy. The latter option bears a resemblance to the “what-the hell” effect—that is, once people cave to lying, they lie by a lot (Mazar et al., 2008; Mead et al., 2009; Ariely, 2012). We test these two possibilities.

A recent paper provides initial support for our argument that a one-by-one setting should lead to more lying than an all-at-once setting. In this work, Schurr et al. (2012) examined the effect of different choice procedures on dishonesty. In one of their experiments participants played a 20-questions trivia game. However, instead of answering the questions, participants were first shown the correct answer and then asked to report whether that was the answer they had in mind. Participants earned money every time they reported they had the correct answer in mind. Obviously, participants could lie to earn more money, and the experimenters had no way to detect lies or liars. Importantly, participants earned more money for difficult questions [e.g., Samuel Langhorne Clemens is better known as: (a) Rudyard Kipling; (b) Edgar Allan Poe; (c) *Mark Twain*; (d) Oscar Wilde] and less money for easy questions [e.g., “The Portrait of Dorian Gray” is a novel by: (a) Rudyard Kipling; (b) Edgar Allan Poe; (c) Mark Twain; or (d) *Oscar Wilde*]. In one condition, participants were asked to choose between an easy and a difficult question before each trial. In another condition, participants decided ahead of time on the number of easy and difficult questions they wanted to solve in 20 trials. When facing a sequence of 20 temptations to cheat (as compared to a single temptation), participants caved in and chose more difficult (i.e., profitable) questions, to which they frequently reported they had the correct answer in mind. Note, however, that in both conditions, the task was identical (answering a sequence of trivia questions). Thus, rather than comparing the effect of reporting policies, this study primarily examined the effect of one planned choice vs. repeated ongoing choices on ethicality.

We now turn to report two surveys and an experiment that aimed to answer the question: Which reporting policy is more effective in encouraging honest self-reports—the one-by-one or the all-at-once?

The surveys tested people's intuitions regarding the two reporting policies. Participants of the first survey thought that the macro-management all-at-once reporting policy conveys more trust from the organization than the micro-management one-by-one policy. Independent of that trust, other participants completing the second survey expected the levels of dishonesty employees will engage in should be the same for the two reporting policies. That is, whereas people perceived the all-at-once policy to demonstrate higher level of trust in employees from the organization's perspective, this trust does not translates to people's prediction regarding employees' likelihood to behave unethically. Next, we report an experiment providing a direct comparison between the one-by-one and all-at-once reporting policies. The experimental task simulated employees self-reporting their performance to their employer for payment. The experimental findings indicate the one-by-one policy led to more cheating than the all-at-once policy. Implications for future research and for organization policy are discussed.

## People's intuitions

As a first step, we assessed the extent to which people have a clear and consensual intuitions regarding the one-by-one and all-at-once reporting policies. This is important as policies are designed based on what people believe the state of the world is. In the first survey, we asked a group of participants which of the two policies conveys more trust in employees' honesty. In a second survey we employed we asked a different group of participants whether employees are more likely to cheat in one of the two policies.

### Intuition regarding trust

#### Materials and method

For this survey, we recruited 93 participants to complete a paid online questionnaire via the Amazon Mechanical Turk website (46 females, *M*_age_ = 36.18, *SD*_age_ = 10.41).

The questionnaire described two fictional companies: Company A utilizes a one-by-one reporting policy and requires regular itemized reports of performance. Company B utilizes an all-at-once reporting policy requiring an integrated overall report of performance. Referring to five common types of performance (working hours, travel expenses, overtime, work progress, and calling in sick), we asked participants to state which of the two companies is more likely to trust their employees to report honestly. For example, the question regarding travel expenses read: “Company A requires their employees to report each of their travel expenses (food, hotels, taxis etc.) on separate forms. Company B requires their employees to report all their travel expenses (food, hotels, taxis, etc.) on just one form.” For each question, participants chose one of three responses that read: “[1] Employees in Company A are more trusted by the organization to report honestly; [2] Employees in Company B are more trusted by the organization to report honestly; [3] Employees in Companies A and B are trusted to report honestly to the same extent by their organizations.”

#### Results and discussion

**Figure 1:**
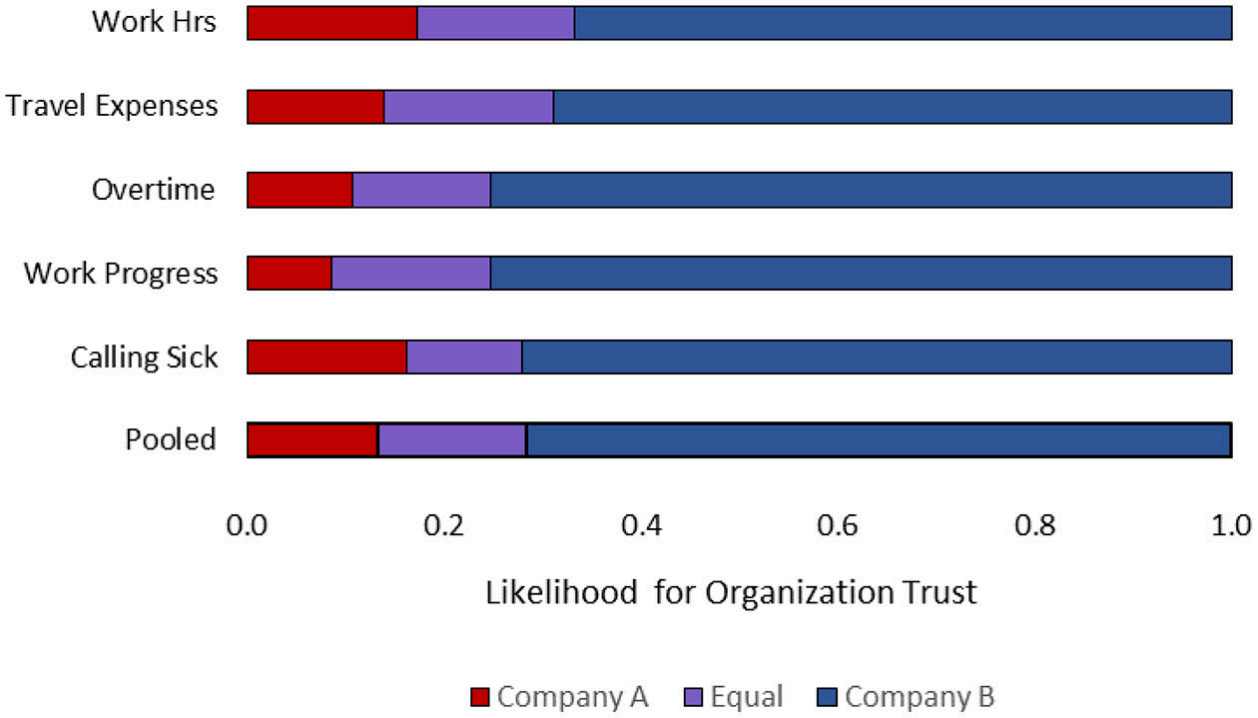
**Relative frequencies of participants' ascribed organizational trust in employees per company**.

The relative frequencies distributions for each of the five questions showed that most participants felt that the all-at-once reporting policy (of Company B) conveys more trust than the one-by-one reporting policy (of Company A). A pooled distribution summarizes the general intuition (see Figure 1). A small proportion of the participants (13%) thought that the one-by-one policy of company A reflect more trust in the employees, a small proportion of participants (15%) thought both policies reflect the same level of trust, and the vast majority of the participants (72%) stated that the all-at-once reporting policy reflects more trust in the employees. We used effect coding (−1 = trust is more likely in Company A; 0 = trust is equally likely in both companies; 1 = trust is more likely in Company B). In line with the frequency distribution, the average of the pooled responses was significantly greater than zero (*M* = 0.59, *SD* = 0.71) *t*_(92)_ = 8.02 *p* < 0.0001.

### Intuition regarding cheating

#### Materials and methods

For a second survey, we recruited an additional 102 participants to complete a paid online questionnaire via the Amazon Mechanical Turk website (51 females, *M*_age_ = 35.17, *SD*_age_ = 11.91).

The survey was identical to the one described above, but this time we asked participants to state in which of the two companies employees are more likely to inflate self-reports. Participants chose one of three responses that read: “[1] Employees in Company A are more likely to inflate their reports; [2] Employees in Company B are more likely to inflate their reports; [3] Employees in Companies A and B are as likely to inflate their reports.”

#### Results and discussion

**Figure 2:**
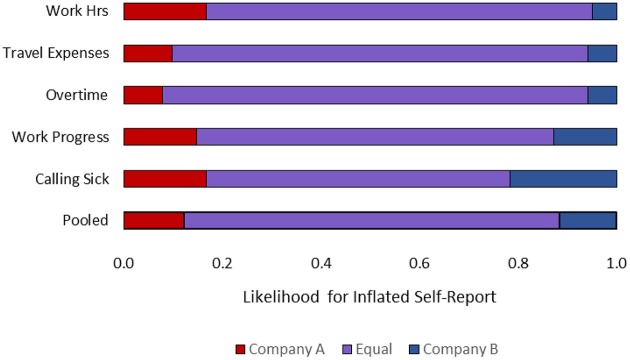
**Relative frequencies of participants' intuitions regarding the effect of reporting policy on inflated self-reports in the workplace**.

The relative frequencies distributions for each of the five questions showed that most participants expected inflated self-reports to be equally likely whether the reporting policy was one-by-one (i.e., Company A) or all-at-once (i.e., Company B). A pooled distribution summarizes the general intuition (see Figure 2). A small proportion of the participants (13%) predicted that the one-by-one policy of company A would lead to more cheating, a small proportion of participants (10%) thought the all-at-once policy would lead to more cheating, and the vast majority of the participants (77%) stated that the reporting policy would not influence the rate of inflated self-reports. We used effect coding (−1 = cheating is more likely in Company A; 0 = cheating is equally likely in both companies; 1 = cheating is more likely in Company B). In line with the frequency distribution, the average of the pooled responses was practically zero (*M* = 0.03, *SD* = 0.47) *t*_(101)_ = 0.62 *p* = 0.534.

Thus, whereas participants perceived the macro-management all-at-once policy as more trusting than the micro-management one-by-one policy, this intuition did not translate to assuming that people will lie more in the one-by-one policy. We next report an experiment that compared the effect of the two reporting policies on actual cheating behavior in a controlled laboratory experiment.

## Experiment

The experiment reported below provides a direct comparison of the one-by-one and all-at-once reporting policies. The experiment was designed to test if the different procedures of the one-by-one and all-at-once reporting policies affect the level of dishonesty.

### Eliciting cheating with a trivia game

To simulate a work setting and allow participants to earn money solely on the basis of self-reports, we adapted the trivia game paradigm (Schurr et al., 2012). The game included two rounds of 20 questions each^1^. The first round was entitled “practice” and did not involve incentives. The second round was entitled “test” and involved incentives. In the “test” round, participants earned a fixed payment each time they stated they had the correct answer in mind. Note that performance level could not improve between the two rounds (i.e., trivia games are based on existing knowledge and each question was presented only once). Thus, the practice round served to establish baseline performance,^2^ and any improvement in the test round represented participants' inflating performance to earn more money. To simulate the two reporting policies, in the one-by-one condition, we presented participants with a series of 20 separate trials. In each trial, participants answered a single trivia question. In the all-at-once condition, we presented participants with a list of 20 trivia questions in a single trial. Participants solved all the 20 trivia questions in this one trial and submitted a 20-line report of their performance (see Appendix 1 in Supplementary Material).

### Materials and methods

We recruited 96 participants (43 females, *M*_age_ = 23.53, *SD*_age_ = 4.89) via an online web system to participate in an experiment at the Cologne Laboratory of Economic Research. We compensated participants with a fixed show-up fee of €5 and an added bonus contingent on their earnings in the test round of the trivia task (€0–€4). The experiment lasted about 30 min.

Upon arrival, participants were seated in computer cubicles that ensured privacy and were randomly allocated to one of the two reporting-policy conditions. In the one-by-one condition, in each trial, a single question was displayed on screen. Participants were asked to think about the answer, keep it in mind, and click a button to reveal the correct one. Participants then clicked a Yes/No button to report whether the answer they had in mind was correct. Participants repeated the same procedure for each of the 20 questions (see Appendix 1 in Supplementary Material). In the all-at-once condition, a list of all 20 questions appeared on the screen. Participants worked through the list (in any order they saw fit). For each question, participants were asked to think about the answer, click a button to reveal the correct one, and then click the Yes/No button to report whether they had the correct answer in mind. Participants could edit and change their responses before they submitted their overall reports (see Appendix 1 in Supplementary Material). In each condition, participants first completed a practice round (without incentives) and then completed a test round (with incentives). In the test round, participants earned €0.2 each time they reported they had the correct answer in mind.

### Results and discussion

The critical measure was the difference in reported performance between the test and practice rounds. As explained above, any improvement from the baseline performance represented cheating for monetary profit.

The findings showed that in both conditions, participants tended to inflate their performance in the test round to earn money. Importantly, participants were much more likely to inflate their performance reports in the one-by-one condition (improving by 10.74%) than in the all-at-once condition (improving by 3.6%, see Table 1).

**Table 1:** **Self-reported performance on the trivia game**.

	***N* (% female)**	**Average correct answers reported**		**“Improvement” average difference**
		**Practice phase**	**Test phase**	
One-by-one	47 (53%)	14.06	15.57	1.51^*^ (*SD* = 2.62)
All-at-once	49 (41%)	14.65	15.18	0.53 (*SD* = 2.22)

* *The mean difference was significantly different from zero at the p < 0.01 level on the two-tailed Wilcoxon signed rank test*.

We submitted the overall performance reports in the practice and test rounds to a repeated measures ANOVA with reporting policy as a between-subject variable. The effect of Round was significant *F*_(1, 94)_ = 16.96, *p* < 0.0001 partial η^2^ = 0.15. The main effect of Policy was not significant *F*_(1, 94)_ < 0, *ns*. The interaction between Round and Policy was marginally significant *F*_(1, 94)_ = 3.91, *p* = 0.051, partial η^2^ = 0.04 indicating that in general participants tended to inflate performance more in the one-by-one condition than in the all-at-once condition. Simple effects analysis revealed significant improvement (compared to the null hypothesis that assumes no improvement) in the one-by-one [*t*_(46)_ = 3.95, *p* < 0.0001] but not in the all-at-once setting [*t*_(48)_ = 1.67, *p* = 0.105].

We further tested the effect using non-parametric tests. The baseline performance in the practice round differed slightly between the two experimental conditions, but the difference was not significant (*Z* = 1.02, *p* = 0.307, Mann-Whitney *U*-test). Participants' self-reports indicated improved (i.e., inflated) performance in the test round. A Wilcoxon signed rank test showed that this improvement was significant in the one-by-one condition (*Z* = 3.473, *p* = 0.0005), but was not significant in the all-at-once condition (*Z* = 1.51, *p* = 0.130). A direct comparison between the two experimental conditions indicated that improvement (i.e., inflated performance) was almost three times larger in the one-by-one condition than in the all-at-once condition (*Z* = 2.14, *p* = 0.032, Mann-Whitney *U*-test).

Thus, in line with lie aversion, the findings showed that the one-by-one policy led to more cheating, whereas the all-at-once policy resulted in considerable self-restraint.

In a follow up analysis we examined behavior at the individual level. Whereas, our design does not allow to determine if a certain participant lied or not, the “improvement” that a participant reports allows us to assess the likelihood of dishonesty. Specifically, in the Trivia paradigm, if a participant reports the same level of performance in the practice and test rounds, there is high likelihood of honesty. Negative improvement scores (i.e., lower performance in the test round compared to practice) suggest that honesty was chosen over the possibility to earn money (and at the personal cost of admitting ignorance). In contrast, reporting better performance in the test round, suggests it is likely that performance was falsely inflated to earn more money.

**Figure 3:**
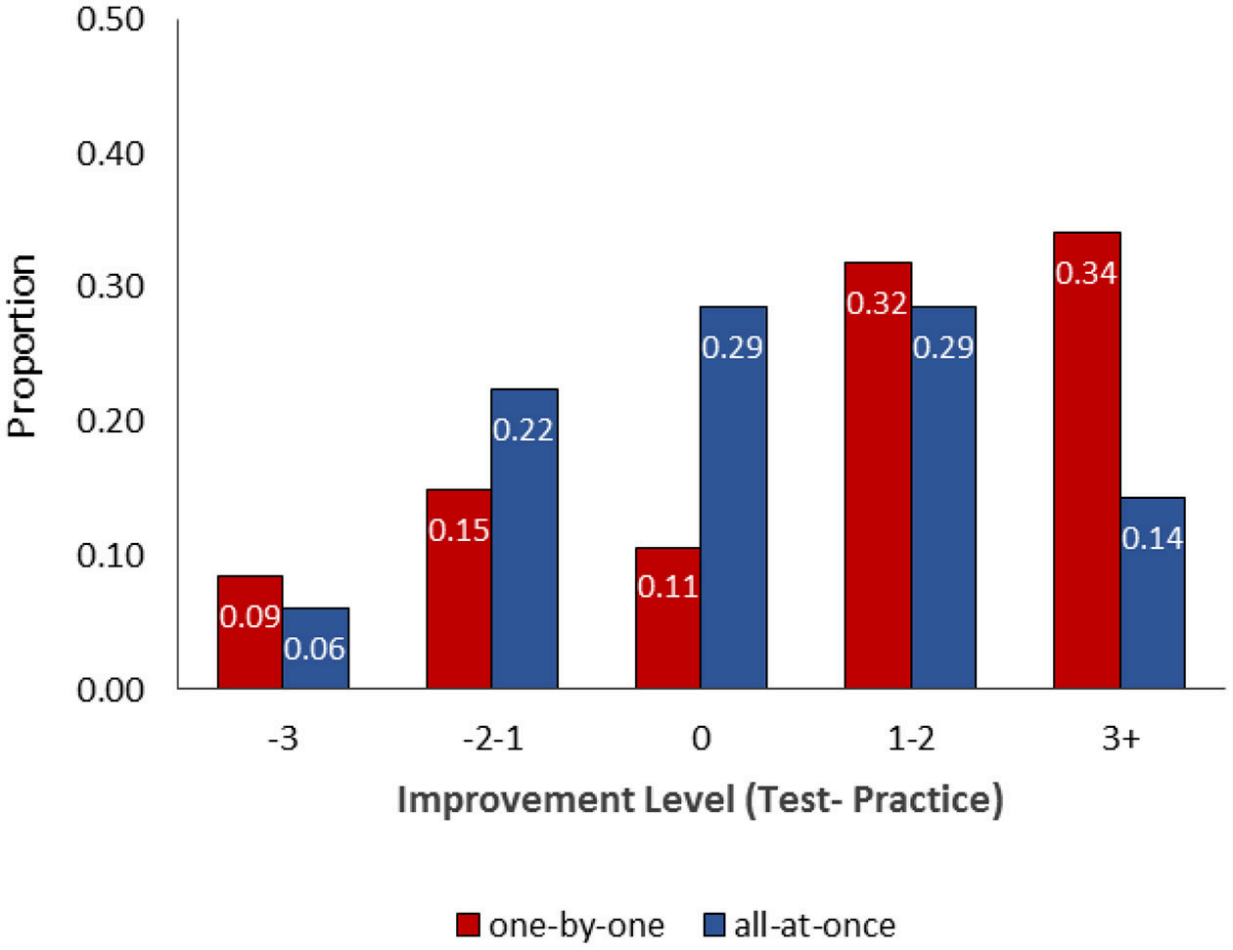
**Frequency distributions of participants' “improvement” scores in the one-by-one and all-at-once reporting policies**.

Figure 3 shows the relative frequency distributions of “improvement” scores in the two experimental conditions. As can be seen, negative to zero improvement scores (i.e., high likelihood of honesty) were more frequent in the all-at-once reporting policy. In contrast, high improvement scores (i.e., high likelihood of dishonesty) were more than twice as likely in the one-by-one policy. The difference between the frequency distributions was marginally significant $\chi_{\left(4\right)}^{2}=8.81$, *p* = 0.066. The pattern lends further support to the idea that one-by-one reporting policy is more likely to facilitate dishonesty than all-at-once reporting policy.

## General discussion

Self-reporting policies aim to apply organizational monitoring and encourage trust at the same time. Here, we compared two specific reporting policies. One policy, entitled one-by-one, requires employees to report separate segments of their performance. Another policy, entitled all-at-once, allows employees to submit an integrated overall report. Objectively, employees could exploit both policies and inflate self-reports to the same extent. Indeed, two surveys revealed that while people perceive the all-at-once policy as more trusting, they still expected people would be equally likely to cheat in both policies. Our results demonstrate however that people lie more in a one-by-one procedure than in an all-at-once procedure.

An analysis at the individual level indicated that participants were more likely to resist temptation and be honest when they were asked to provide a single report of their performance. In contrast, participants were more likely to inflate performance to a large extent when they provided a sequence of segmented reports. The finding is in line with the idea that repeated reports in the one-by-one policy make it harder for people to resist the temptation to cheat, and that once they cave in to the temptation to lie, they lie by a lot.

As always, generalization of experimental findings should be cautious, and subject to the accumulation of research that offers replications of the effect as well as identifying boundary conditions. For example, Desai and Kouchaki (2015) recently reported a study that seemed to favor the one-by-one policy over the all-at-once policy. In this study, an experimenter contacted garage mechanics to obtain an estimate for changing the brake pads of a car. The findings showed that in this set-up, mechanics inflated costs less when they provided separate estimates for two different aspects of the job (i.e., parts, labor), than when they provided an overall cost estimate. The authors offered that specific estimates elicited higher accountability requiring mechanics to be able to justify their quotes (Desai and Kouchaki, 2015, study 7). It is difficult to establish a direct comparison between offering a price quote to a client and reporting performance to an employer. Still, the finding raises an interesting question regarding a potential difference between one-time task and repeated tasks. For example, what would have happened, if the mechanics had to provide price quotes repeatedly to more clients? Would the segmented quote still be lower than the overall quote for the 10th and 20th clients? This question sets an interesting direction for future research.

On a theoretical level, one-by-one and all-at-once reporting procedures can be used to test possible interactions between accountability (e.g., Tetlock, 1992; Lerner and Tetlock, 1999; Desai and Kouchaki, 2015) and lie aversion (e.g., Gneezy, 2005; Lundquist et al., 2009; Shalvi et al., 2011b; Hilbig and Hessler, 2013). The two constructs could probably be combined to work in the same direction and encourage honesty. In the setting we examined, however, we suspect that accountability and lie aversion may have operated against each other. To wit, the one-by-one itemized report may have elicited a sense of accountability and increased the need to justify one's actions. Note however, that in our experiment, such reporting procedure involved small lies that disappeared from the screen at the end of each trial. The combination of an increased need to justify one's action and small lies that are easily forgotten may have led to a paradoxical outcome that minimized the psychological cost of guilt and facilitated cheating. More research is needed to examine and fully establish the ways in which lie aversion and accountability interact, as well as the contextual factors that may determine their joint effect.

It is worth noting that the experimental task we employed provides only direct measures of self-reported improvement rather than explicit measures of cheating. While we assume that improvement in the trivia tasks points at high likelihood of cheating—it is of course possible that some participants indeed improved in their performance levels in the test round. We chose this experimental task because it has high external validity (as people often do not know if a person is cheating or not, just receive indirect indications for such possibility). Future research may benefit from experimental tasks that allow to trace individual's dishonesty more directly. Such future work can further explore if individual differences in relevant parameters such as gender, moral disengagement, or moral identity, may moderate any of the observed effects. This would increase our understanding of the patterns identified here.

On an applied note, the findings are also important for the development of organizational monitoring policies that aim to prompt both honesty and trust. Indeed, in a survey we found that people consider organizations implementing an all-at-once policy to be more trusting of their employees' honesty than organizations implementing the one-by-one policy. Our experiment suggests that close monitoring policy might lead people to discount segmented transgressions as minor and negligible and thus result in lower honesty levels. Our findings offer an optimistic view showing that the more trusting policy led to more honest behavior.

Many organizational activities can be categorized as one-by-one or all-at-once tasks. For example, many people sort out their incoming emails by automatically placing them in multiple folders, which in turn get packed with many items waiting to be dealt with. Does the number of folders affect people's responses to those emails? People also use reporting systems to report different purchase orders, submit expenses reports, tax reports, and so forth. Our findings suggest that the effect of reporting procedure on honesty is not trivial and policy makers should consider carefully the reporting procedures and even the display format of reporting systems to design settings that encourage ethical conduct.

## Ethic statement

All studies were approved by the Institutional Review Boards at the university of Cologne. All participants read and signed an informed consent before the studies.

## Author contributions

All authors listed, have made substantial, direct and intellectual contribution to the work, and approved it for publication.

### Conflict of interest statement

The authors declare that the research was conducted in the absence of any commercial or financial relationships that could be construed as a potential conflict of interest.

## Footnotes

^1^We selected 40 trivia questions for the practice and test rounds from a pool of 200 questions that were pretested. The level of difficulty was controlled and was comparable within and between rounds.

^2^One reason to inflate performance has to do with impression management and social desirability, in that no one wants to admit ignorance. Thus, the baseline performance that participants report in the practice round reflects a composite of their true performance and self-enhancement. The incentives in the test round present an additional temptation to cheat—this time for monetary profit. Thus, any “improvement” in the second round isolates the component of dishonesty that is harnessed to increase monetary profit.
