# Supplementary material for: One-by-One or All-at-Once? Self-Reporting Policies and Dishonesty
Source: Front Psychol. 2016 Feb 17;7:113. doi: 10.3389/fpsyg.2016.00113 (PMC4756120; doi:10.3389/fpsyg.2016.00113)
Supplement: Supplementary file 1 [file DataSheet1.docx]

**Appendix 1**

**Schematic examples of the displays in the one-by-one and the all-at-once conditions**

Sample item in a one-by-one trial

| Question 1 | Click to reveal Answer | | Did you have the correct answer in mind? |
| --- | --- | --- | --- |
| The character portrayed by Russell Crowe in the film A Beautiful Mind won the Nobel Prize in: | [ a ] Economics  [ b ] Physics | ******* | [x] Yes  [x] No  Click to submit |
|  |  |  | |

Sample items in an all-at-once trial

| Question 1 | Click to reveal Answer | | Did you have the correct answer in mind? |
| --- | --- | --- | --- |
| The character portrayed by Russell Crowe in the film A Beautiful Mind won the Nobel Prize in: | [ a ] Economics  [ b ] Physics | ******* | [x] Yes  [x] No |
| Question 2 | Click to reveal Answer | | Did you have the correct answer in mind? |
| The volcano Nevado del Ruiz that erupted in 1985 and caused major loss of life is located in: | [ a ] Bolivia  [ b ] Columbia | ******* | [x] Yes  [x] No |
| . . . . . . . . . . . . . . . . . . . . . . . . . . . . . . . . . . . . . . . . . . . . . . . . . . . . . . . . . . . . . . . . . . . . . . . . . . . . . . . . . . . . . . . . . . | | | |
| . . . . . . . . . . . . . . . . . . . . . . . . . . . . . . . . . . . . . . . . . . . . . . . . . . . . . . . . . . . . . . . . . . . . . . . . . . . . . . . . . . . . . . . . . . | | | |
| Question 18 | Click to reveal Answer | | Did you have the correct answer in mind? |
| In 2007, Brian May, a founding member of Queen and a world-renowned guitarist, completed his doctorate in: | [ a ] Astrophysics  [ b ] Psychology | ******* | [x] Yes  [x] No |
| Question 19 | Click to reveal Answer | | Did you have the correct answer in mind? |
| Epistaxis is: | [ a ] Ringing in the ears  [ b ] Nosebleed | ******* | [x] Yes  [x] No |
| Question 20 | Click to reveal Answer | | Did you have the correct answer in mind? |
| An anemometer measures: | [ a ] Wind-speed  [ b ] Air-pressure | ******* | [x] Yes  [x] No |
|  |  | Click to submit | |
